# Comparative Genomic Analysis of Bovine and Publicly Available Human *Streptococcus agalactiae* Genomes

**DOI:** 10.3390/ani16142257

**Published:** 2026-07-21

**Authors:** Gabriele Meroni, Valerio Massimo Sora, Alessio Soggiu, Piera Anna Martino, Giulia Laterza, Luciana Colombo, Francesca Zaghen, Luigi Bonizzi, Alfonso Zecconi

**Affiliations:** 1 One Health Unit, Department of Biomedical, Surgical and Dental Sciences, School of Medicine, University of Milano, Via Pascal 36, 20133 Milan, Italy; alessio.soggiu@unimi.it (A.S.); piera.martino@unimi.it (P.A.M.); giulia.laterza@unimi.it (G.L.); luigi.bonizzi@unimi.it (L.B.); alfonso.zecconi@unimi.it (A.Z.); 2 Associazione Regionale Allevatori Lombardia, Via Kennedy 30, 26013 Crema, Italy; l.colombo@aral.lom.it (L.C.);

**Keywords:** *Streptococcus agalactiae*, whole genome sequencing, bovine, pan-genome, antimicrobial resistance, virulence

## Abstract

Background/Objectives: *Streptococcus agalactiae* is one of the most significant pathogens causing infections in humans and mastitis in dairy cattle. This work focused on a comprehensive comparative pan-genomic analysis of bovine and human Group B *Streptococcus* to elucidate the genetic mechanisms underlying host adaptation and dissemination. Methods: Isolates of *S. agalactiae* from quarter milk samples from dairy herds in Lombardy (Italy), along with human strains, were considered. Whole genome sequencing was used to compare core and accessory genomes, assign sequence types, and find virulence and resistance factors. Results: 30 sequence types were detected, of which two (ST12 and ST23) in common between bovine and human. The allele frequencies for resistance determinants revealed elevated rates for *tetM* (59.8% overall, 66.5% in human clinical isolates), *ermB* (17.3% overall, 20.4% in human clinical isolates), and *ant(6)la* (8.9% overall, 12.8% in human clinical isolates). Bovine strains had accessory gene clusters linked to lactose metabolism and immunological evasion, whereas human isolates were concentrated in regions related to adhesion and antibiotic resistance. Conclusions: Comparative pan-genomics show that there is a small genetic overlap between bovine and human Group B *Streptococcus* populations.

## Simple Summary

Group B *Streptococcus* (*Streptococcus agalactiae*) can cause serious disease in newborns and adults, and it is also a major cause of mastitis in dairy cows. In this study, we compared the complete genetic backbone of bacteria isolated from human patients and from cow milk on dairy farms in Lombardy (Italy). By analyzing thousands of genes, we identified which groups of strains are shared between humans and animals and which are specific to one host. We show that some bacterial lineages and capsule types are strongly adapted to either humans or cows, while a smaller number appears in both, suggesting possible similarities of bacteria between species. We also examined genes and predicted traits linked to antibiotic resistance and disease severity. Many strains from both hosts carried markers of resistance to important antibiotics, and some human and bovine strains showed very similar resistance patterns, raising concerns about the spread of these hard-to-treat lineages. Some human and bovine strains showed overlapping resistance profiles and genomic features, supporting the One Health relevance of comparative surveillance. However, these data indicate genetic similarity and shared traits rather than direct evidence of recent transmission between hosts.

## 1. Introduction

The transmission of *Streptococcus agalactiae* (Group B *Streptococcus*, GBS) between cattle and humans remains a critical area of research in veterinary and medical microbiology from a One Health perspective. *S. agalactiae* is recognized worldwide as a primary pathogen in cattle, particularly associated with bovine mastitis. As an example, the prevalence of *S. agalactiae* positive herds in the Lombardy region (Italy) showed a prevalence of 7.3%, confirming the presence of these pathogens in dairy herds and the need to eradicate them for health and economic reasons [1,2]. *Streptococcus agalactiae* is also affecting human beings, especially in neonatal and immunocompromised groups, and GBS is a predominant cause of serious infections, such as sepsis and meningitis [3,4]. For such reasons, comprehending the transmission dynamics is essential for developing effective prevention and control strategies. The epidemiology of GBS is complex, with different sequence types (ST) showing several virulence determinants and host affinities. Notably, ST103 and ST591 have been recognised as common in both bovine and human populations, indicating a possibility for interspecies transmission that may affect GBS pathogenicity and antibiotic resistance characteristics [5,6,7]. Genomic analyses on different GBS strains from various countries indicated that many human-derived strains and clinical isolates were grouped into five principal clonal complexes (CC1, CC10, CC17, CC19, and CC23) [8], while bovine-derived ones were classified under a unique clonal complex, CC67 [9]. Exposure to cattle showed to be a predictor of human colonisation with *S. agalactiae*, according to a prospective cross-sectional cohort study [10]. Case reports suggest potential transmission between human hosts and cattle and between human hosts and both canines and felines [11,12,13]. Furthermore, experimental investigations have shown evidence of transfer of bovine and human *S. agalactiae* strains to fish [14,15,16]. Nonetheless, information about interspecies transmission is limited, and research on strain typing including a wide array of hosts and geographical regions are deficient. In Denmark, 71% of *S. agalactiae* isolated from bulk tank milk was classified as ST1, ST23, or ST103 [17]. ST1 and ST23 are host generalists frequently identified in humans. ST23 is also recurrent in many animals, aquatic mammals, and ectothermic species, but ST103 is predominantly associated with bovine milk [18,19]. The presence of these pathogens in dairy herds, the related costs, and the potential transmission to humans, support the need to eradicate the microorganism in dairy herds [1,2]. However, the success of an eradication program is affected by the circulation of the pathogens among species different from the ones targeted by the eradication program itself (dairy cows). Therefore, the potential ability of *S. agalactiae* to infect both humans and cows represents a major problem in a One Health perspective, but also a factor hampering the success of an eradication program. Previous studies have suggested host adaptation and possible interspecies transmission of *Streptococcus agalactiae*, but most evidence is based on strain typing, case reports, or selected genomic markers. Therefore, the broader pan-genomic differences and shared genomic features between bovine and human populations remain insufficiently defined. In pan-genome analyses, gene families are commonly classified according to their frequency across the analyzed genomes. Core genes are present in nearly all isolates (95–100% of all isolates) and represent the conserved genomic backbone of the species. Shell genes occur at intermediate frequencies (15–95% of all isolates) and usually represent accessory functions shared by subsets of strains, whereas cloud genes are rare genes found in only a few genomes (0–15% of all isolates) and often reflect highly variable accessory content. The objective of this study was to compare bovine and human *S. agalactiae* genomes through a harmonized pan-genomic, resistome, and virulome analysis, to identify host-associated genomic signatures, shared lineages, and overlapping AMR/virulence profiles relevant to One Health surveillance.

## 2. Materials and Methods

### 2.1. Inclusion Criteria and Sampling

The dataset included 100 *S. agalactiae* genomes: (50 bovine isolates and 50 human clinical genomes); bovine isolates were obtained from quarter milk samples collected from dairy herds during a one-year sampling period. Moreover, to minimize clustering due to multiple isolates from the same epidemiological unit, only one isolate per herd was included in the genomic comparison and for this reason the bovine dataset should be interpreted as a herd-level, non-duplicated collection. Human genomes were retrieved from public genomic databases and included only when they were assigned to *S. agalactiae*, derived from human clinical samples, associated with an accession number, and accompanied by sufficient metadata for host origin and geographic attribution. Italian human clinical genomes were prioritized; when the number of available Italian genomes was insufficient to reach the predefined sample size, European human clinical genomes were included. In total, 35 human genomes originated from Italy and 15 from other European countries. Publicly available genomes were not generated within the present study and may therefore differ from the bovine genomes in sampling design, sequencing platform, and metadata completeness; to minimize technical heterogeneity between newly sequenced bovine genomes and publicly available human genomes, all genomes included in the comparative analyses were processed through a harmonized downstream workflow. Public genomes were included only when assemblies were available with sufficient metadata on host origin, geographic origin, and accession number and genome quality was assessed before inclusion. The same annotation, pan-genome, AMR, and virulence-screening procedures were then applied to both bovine and human genomes to reduce annotation- and database-related biases.

### 2.2. Bacterial Isolation and Identification

Aseptically collected, quarter milk samples from dairy cows were utilized for the isolation and identification of *S. agalactiae*, following the protocols and recommendations established by the National Mastitis Council [20]. The initial identification relied on Gram staining, followed by catalase test; then, pure colonies were subcultured in Brain Hearth Infusion Broth (BHI, Microbiol, Uta, Italy) and incubated at 35–37 °C for 24–48 h under microaerophilic atmosphere and analyzed using Vitek instrument (BioMerieux, Lyon, France) to confirm the preliminary identification. The next day, 750 µL of broth were diluted with an equal volume of sterile glycerol (50% *v*/*v*) and stored at −20 °C for further use. The day before the experiment, 50 µL of stock cultures were plated on Tryptic Soy Agar (TSA, Microbiol, Uta, Italy) supplemented with 5% defibrinated sheep blood and incubated at 35–37 °C for 24 h in microaerophilic atmosphere to have fresh overnight cultures.

### 2.3. DNA Extraction

Isolated colonies from a fresh overnight culture of *S. agalactiae* on TSA agar + 5% sheep blood were resuspended in 500 μL of PBS (Phosphate Buffered Saline, Euroclone, Milan, Italy) and centrifuged at 10,000× *g* for 1 min at room temperature. DNA was extracted in accordance with the manufacturer’s instructions using the Quick-DNA™ HMW MagBead kit (Zymo Research, Irvine, CA, USA).

The A_260_/A_280_ and A_260_/A_230_ ratios were confirmed to be within the range of 1.8 to 2 by evaluating the quantity and quality of the extracted DNA using UV-Vis spectrophotometry with the NanoReady Touch Micro Volume Reader (Aurogene, Rome, Italy).

### 2.4. Sequencing and Bioinformatics Pipelines

Genomic libraries were generated by tagmenting 200 ng of input DNA per sample with transposase using the Rapid Barcoding Sequencing kit (SQK-RBK114.24, Oxford Nanopore Technologies, Oxford, UK). Twelve strains were subsequently transferred onto a single flowcell (FLO-MIN114, version R10.4.1, Oxford Nanopore Technologies, Oxford, UK) and sequenced for a maximum of 72 h using the third-generation MinION Mk1C sequencer (Oxford Nanopore Technologies, Oxford, UK).

Dorado (v0.8.2) [21] was used for demultiplexing, adapter trimming, and basecalling. NanoPlot (v1.44.0) [22] generated summary data regarding the quantity and quality of the reads. FiltLong (version 0.2.1) [23] was employed to conduct the filtration with a minimum cut-off of 1 kbp. The genomes were subsequently de novo assembled using Flye (v2.8.1-b1676) [24], and the resulting contigs were refined and corrected using Medaka (v2.0.1) [25]. CheckM2 (v1.2.4) [26] evaluated the genomic contamination and completeness. For comparative analyses, both newly generated bovine assemblies and publicly available human assemblies were subjected to the same downstream bioinformatic workflow using the same software versions, reference databases, and identity/coverage thresholds. This strategy was adopted to reduce technical variation due to differences in original sequencing platform or assembly source.

After genome annotation with Prokka, several complementary pangenome and gene content pipelines were applied. PPanGGOLiN (v2.2.6) [27] was used to infer the open pangenome, assign gene families to persistent, shell and cloud partitions, and delineate regions of genome plasticity and associated modules across isolates. Anvi’o (v8) [28] was employed to generate annotated contig databases, compute genomewide average nucleotide identity where required, and visualise gene presence–absence patterns and functional enrichment along the genome. In parallel, a second pangenome was built with Roary (v3.13.0) [29] using a 95% BLASTp v2.16.0 identity threshold and a ≥99% core definition to provide an independent estimate of core and accessory gene sets. A core-gene nucleotide alignment was produced by Roary using MAFFT (-e --mafft) (v7.526), resulting in a concatenated core-genome alignment for all strains. The concatenated core-genome alignment (core_gene_alignment.aln) was subsequently used to infer a maximum-likelihood phylogeny with FastTree (v2.2), using the nucleotide mode and a general time-reversible–like model with among-site rate heterogeneity (default CAT approximation), which is commonly applied to bacterial core-genome datasets. The resulting tree was midpoint-rooted and visualized with iTOL.

Capsular polysaccharide and surface protein loci were typed with GBSSBG, allowing integration of serotype information with sequence type and pan-genome partitions [30]. Antimicrobial resistance and virulence genes were screened with ABRicate (v1.2.0) [31,32,33] against the CARD and VFDB databases, respectively, retaining hits above curated identity (95%) and coverage (80%) thresholds for downstream mechanism and category-level analyses. In a second step, whole-genome-based predictions of phenotypic susceptibility/resistance were obtained with the ResFinder web tool (version 4.0), using default parameters and the corresponding ResFinder databases, to infer predicted non-susceptibility for a panel of clinically relevant antimicrobials from the detected AMR gene content. These ResFinder-based predictions were used to construct binary AMR profiles at the antibiotic level for multivariate analyses and the minimum-spanning tree. These data represent in silico predictions of acquired AMR determinants and should be interpreted as genotypic indicators of potential non-susceptibility, rather than as confirmed phenotypic resistance profiles.

Finally, functional profiling of the pan-genome was refined with panVITA [34], which was used to annotate and classify protein families into virulence associated categories that were subsequently mapped to STs and PPanGGOLiN partitions.

The genomes of the isolated bacteria were compared to those in a public database (Pathogen Detection, GenBank) to analyze pathogenic features, including the presence of antibiotic resistance genes and clonality.

### 2.5. Statistical Analyses

Analyses were carried out in Linux using Python (v3.14.0). Tabular data were handled with pandas and plotted with matplotlib. Categorical variables (e.g., host, ST, resistance mechanism, virulence category) were encoded as factors; counts and continuous measures (e.g., number of ineffective drugs, gene counts) as integer or float vectors.

Multivariate structure of resistance profiles was examined by principal component analysis (PCA) using sklearn.decomposition.PCA. Binary resistance matrices (genomes × antibiotics) were centred and scaled with StandardScaler, and PCA was performed on the correlation matrix. We kept components with eigenvalues > 1, but focused on the first two, which together explained more than 80% of the variance. Variable contributions were evaluated from loadings (eigenvectors scaled by the square root of eigenvalues), and their robustness checked by simple permutation tests on the resistance matrix (1000 permutations).

Co-occurrence of resistance traits was visualised with UpSet plots. Binary resistance indicators were converted to Boolean form and transformed with indicators to obtain intersection sizes. UpSet was then used to display set sizes (per drug prevalence) and intersection sizes, ordered by frequency, to highlight dominant and rare multidrug patterns.

## 3. Results

### 3.1. Genomic Features of the Analyzed Strains

Supplementary Table S1 reports the principal sequencing characteristics of the animal-associated and human-associated genomes. All the sequences were deposited in National Center for Biotechnology Information (NCBI) under the bioproject PRJNA1298752. The distribution of serotype and sequence type (ST) among animal (A_) and human (H_) isolates of *S. agalactiae* reveals significant host specificity. Human-derived isolates are predominantly characterized by serotype III-2 (38%), followed by serotype V (16%), serotype V-like (4%), Ia (8%), III-1 (8%), II (6%), Ib (6%), and IX (2%). In contrast, A_ isolates are represented by serotype II (70%), followed by III-3 (8%), Ia (8%), III-2 (4%), Ib (2%), and IV (2%). The distribution of sequence types showed that ST17 is the most common among human isolates (22%), followed by not determined strains (18%), and a group of singletons (ST130, ST26, ST82, ST23, ST28, ST1785, ST21, ST121, ST1786, ST1784, ST496, ST467, ST10, ST3, ST1390, ST452, ST196, ST459, ST533, ST19, ST136, ST335, and ST529), each at 2%. In animal isolates, ST591 makes up 66% of the population, not determined strains covers up to 16%, followed by ST103 (8%), while ST380, ST12, ST23, ST486, and ST1363 are all present at 2%.

### 3.2. Pan-Genome Analysis

**Figure 1:**
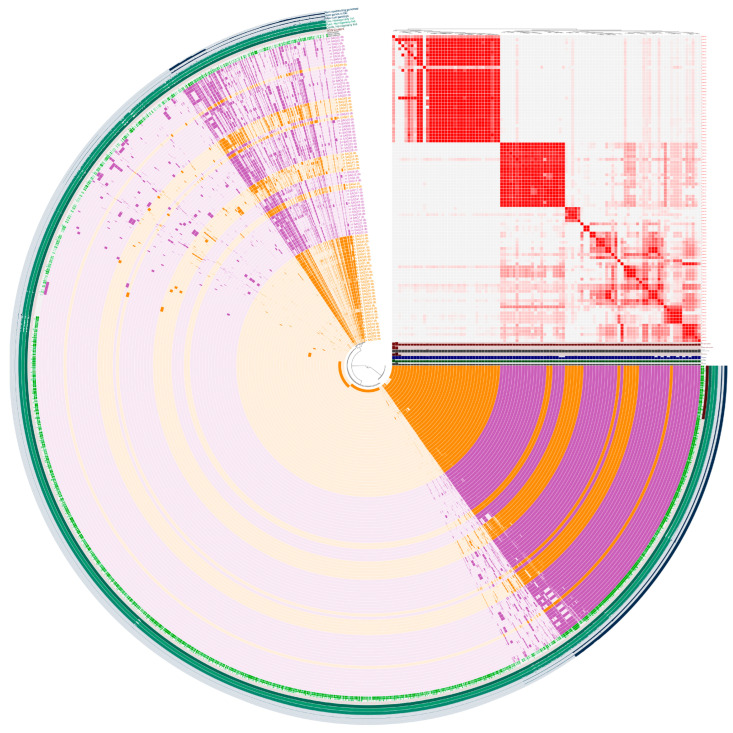
Circular representation of the *Streptococcus agalactiae* pangenome generated with the anvi’o pan-genomics workflow. Each concentric ring corresponds to one genome and each segment represents a gene cluster, ordered along the consensus gene cluster genome; presence is indicated by filled segments, absence by gaps. Strains isolated from animals are highlighted in yellow, whereas strains isolated from humans are shown in purple, illustrating host-associated differences in gene content across the collection.

The open pan-genome structure (Figure 1, Supplementary Table S2) showed a clear separation between animal- and human-derived strains, indicating host-associated genomic structuring within the analyzed collection. However, because the bovine subset was dominated by ST591, these differences should be interpreted as the combined result of host origin and lineage composition rather than as direct evidence of host adaptation alone. The presence of red blocks in the Average Nucleotide Identity (ANI) similarity matrix (99% similarity, top right) indicates the presence of genomic subgroups, likely corresponding to phylogenetic clades. COG20 data analysis allows us to distinguish between known (green) and unknown (gray) categories, functions and pathways, highlighting how a significant portion of the *S. agalactiae* pan-genome remains functionally uncharacterized. Specifically, it is shown the predominance of genes of unknown function among the accessory and unique clusters (visible as gray and white segments). Quantitative data show that the total pan-genome comprises 14,694 genes, divided into four key categories: core genes (present in 99–100% of strains, n = 1109), soft core genes (95–99%, n = 223), shell genes (15–95%, n = 1486), and cloud genes (0–15%, n = 11,876). This distribution reflects a typical structure of highly plastic and adaptable species, where the core fraction represents only a small part of the total genetic repertoire (about 7.5%), while most genes are accessory elements (shell and cloud) that contribute to phenotypic diversity and adaptation to different ecological niches [35].

**Figure 2:**
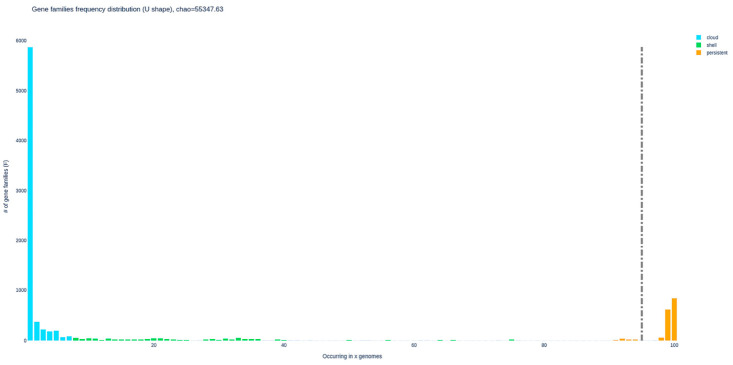
Distribution of gene families across the analysed *S. agalactiae* genomes, showing the characteristic Ushaped pangenome profile. Gene families are grouped according to their partition (cloud, shell, and persistent); most families occur in very few genomes (cloud), while a smaller subset is shared by all or almost all genomes (persistent), consistent with a large and open accessory genome. The dashed grey vertical line indicates the frequency threshold used by PPanGGOLiN to define the persistent genome. The Chao estimator reported in the title reflects the extrapolated size of the pangenome, providing an estimate of the total number of gene families expected in the species beyond those observed in the sampled genomes.

The PPanGGOLiN -generated presence–absence matrix (Supplemental Figure S1) shows that each A_SAG and H_SAG genome encodes ~1540–1580 soft-core families, of which 845 belong to the exact core, highlighting a highly conserved backbone despite marked variability in accessory content. The persistent partition accounts for 1640–1711 genes per genome and remains largely single copy, whereas 19–64 families per genome occur in multicopy within the persistent set, often corresponding to ribosomal components and essential metabolic enzymes. In contrast, the shell compartment ranges from ~300 to >800 genes per genome (e.g., 297 shell families in A_SAG17 versus 847 in A_SAG10), capturing lineage-specific expansions of transporters, regulatory proteins and envelope-associated functions. The cloud partition is particularly inflated in some A_SAG isolates (up to 2660 cloud genes and 60 cloud families in multicopy in A_SAG32), reflecting the presence of large, low-frequency modules that frequently coincide with regions of genome plasticity and are unevenly represented among H_SAG genomes. The U-shaped frequency distribution of gene families (Figure 2) indicates a compact persistent genome, composed of ~1546 soft-core families and 845 exact core families, contrasted with an expansive accessory compartment dominated by low-frequency cloud genes.

On the right tail of the distribution, a relatively small set of families is present in ≥95% of genomes and encodes canonical housekeeping functions, including ribosomal proteins, translation factors, central carbon metabolism, and lipid and cell wall biosynthesis, matching the functional enrichment observed in the persistent partition of the matrix. The left tail comprises thousands of cloud families that occur in one or a few genomes, many of which are organized into accessory modules classified as “cloud”, and frequently encode transport systems, regulatory proteins, mobile elements, and diverse metabolic pathways. Shell families occupy the intermediate frequencies and are overrepresented in “shell” modules with high genome coverage and moderate family counts, which often correspond to regions of genome plasticity and PPanGGOLiN “spots” that recurrently host accessory loci across multiple A_SAG and H_SAG genomes.

### 3.3. Phylogeny

**Figure 3:**
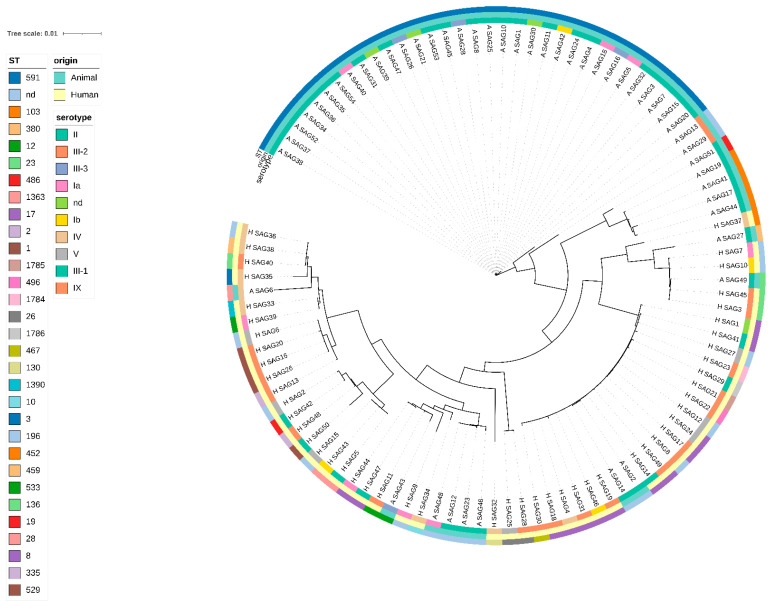
Coregenome phylogeny of *S. agalactiae* isolates. Maximum-likelihood phylogenetic tree inferred from the concatenated alignment of core gene families, showing the relationships among S. agalactiae isolates from animal (A) and human (H) hosts. The outer-colored circles indicate, respectively, the sequence type (ST), the host of origin, and the capsular serotype of each isolate, highlighting the co-occurrence of specific ST–serotype combinations and the clustering of animal- versus human-derived strains within the coregenome diversity. (tree scale: 0.01).

Figure 3 shows the presence of a marked phylogenetic structure, with well-defined clades grouping strains of the same origin. Specifically, several clusters are observed, consisting almost exclusively of animal strains (A_), as well as those composed solely of human strains (H_). Although shared accessory gene families are present across some bovine and human genomes, the current data do not allow recent horizontal gene transfer events to be inferred. Rather, the observed pattern supports host- and lineage-associated genomic structuring within the analyzed collection. This observation is also confirmed by the distribution of the pan-genome; most accessory genes (shell and cloud, which represent over 90% of the total pan-genome) show a non-uniform distribution among strains, contributing to the formation of cohesive and phylogenetically distinct genomic subgroups, as also visible in the red blocks of the similarity matrix.

### 3.4. Antibiotic Resistance of S. agalactiae in Humans and Animals (H_SAG and A_SAG)

**Figure 4:**
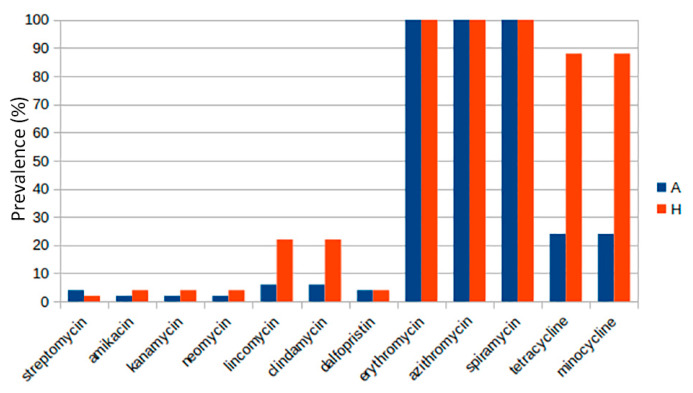
Predicted susceptibility/resistance categories inferred from WGS data using ResFinder 4.0, expressed as prevalence (%) in H_SAG and A_SAG strains.

The WGS-based AMR prediction of S. agalactiae is reported in Figure 4. Gene-level screening (Supplementary Table S3) shows that a large proportion of isolates harbour acquired MLS_B determinants, including *ermB* and *ErmA*/MLS_B methylase genes, as well as *lsaC* and *lnuC*, which are associated with reduced susceptibility to macrolides, lincosamides and streptogramins. Consistent with these findings, ResFinder-derived predictions indicate uniformly high predicted non-susceptibility to macrolides (azithromycin, spiramycin, erythromycin) in both bovine and human isolates, although no phenotypic AST was performed to confirm these predictions. In H_ strains the predicted genotypic resistance exceeds 90%, whereas in animal it ranges from 60% to 70%. The predicted genotypic resistance to other antibiotics, such as aminoglycosides (streptomycin, amikacin, kanamycin), is extremely low (<10%) in both groups. In contrast, lincomycin and dalfopristin exhibit a more pronounced predicted genotypic resistance in human strains, with values that exceed 20%, whereas is nearly absent in animals.

**Figure 5:**
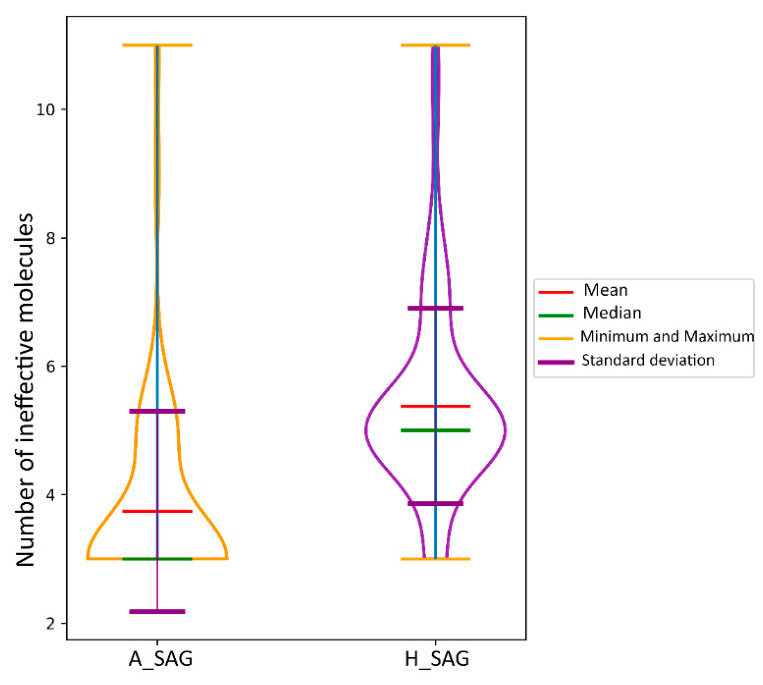
Violin plots of the distribution of WGS-predicted AMR burden in human and animal *S. agalactiae* genomes.

Figure 5 summarizes the per-isolate burden of WGS-predicted AMR, expressed as the number of antimicrobial molecules predicted to be ineffective for each genome. The relevant pattern is not only a difference in median values, but mainly a difference in dispersion. Animal-derived isolates mostly cluster within a limited range of predicted ineffective molecules, whereas human-derived isolates show a broader distribution, with several genomes accumulating additional predicted non-susceptibilities. A small number of bovine isolates also show multidrug profiles comparable to human isolates.

**Figure 6:**
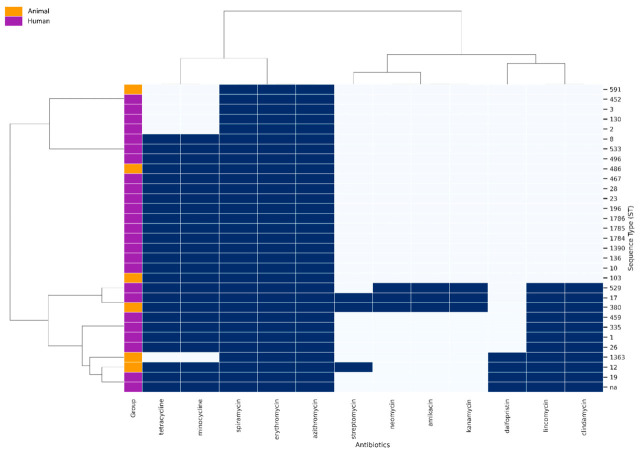
Hierarchical clustering of antibiotic resistance profiles in *S. agalactiae* isolates from animal and human hosts. Heatmap of genotypic predicted resistance to macrolides (spiramycin, erythromycin, azithromycin), tetracyclines (tetracycline, minocycline), lincosamides (lincomycin, clindamycin), streptogramins (dalfopristin), and aminoglycosides (streptomycin, neomycin, amikacin, kanamycin), with isolates annotated by sequence type (ST) and host origin.

The presence of bovine isolates with AMR profiles comparable to human isolates indicates that expanded WGS-predicted AMR profiles can also occur in the animal-derived population, although this observation alone does not demonstrate direct resistance gene transfer. The hierarchical clustering (Figure 6) illustrates the resistance to macrolides (spiramycin, erythromycin, azithromycin) and tetracyclines (tetracycline, minocycline) across nearly all strains, irrespective of their source.

An additional interesting finding is the distribution of resistance to the other antibiotics analysed (Supplemental Figure S2). Resistance to lincomycin, clindamycin, and dalfopristin is considerably more varied and is shown exclusively in some strains, both in animals and humans. Certain A_ ST (orange) exhibit greater resistance to lincomycin, dalfopristin and clindamycin. In human strains (purple), resistance to various antibiotics is often less prevalent, although several clusters exhibit intermediate to high levels. The heatmap indicates typically low resistance to aminoglycosides (streptomycin, neomycin, amikacin, kanamycin). The lateral dendrogram shows that the strains cluster not only by their animal or human origin but also by common resistance patterns. ST1, ST17, ST103, ST196, ST335, and ST452 are present in both animal and human samples, often exhibiting highly analogous resistance patterns, especially against macrolides (spiramycin, erythromycin, azithromycin) and tetracyclines (tetracycline, minocycline). Conversely, several STs seem to be solely represented in one of the two groups. For instance, ST8, ST12, ST19, ST28, ST533, ST496, and ST486 are exclusively found in human samples, but ST380, ST529, and ST1363 are solely present in animal ones.

**Figure 7:**
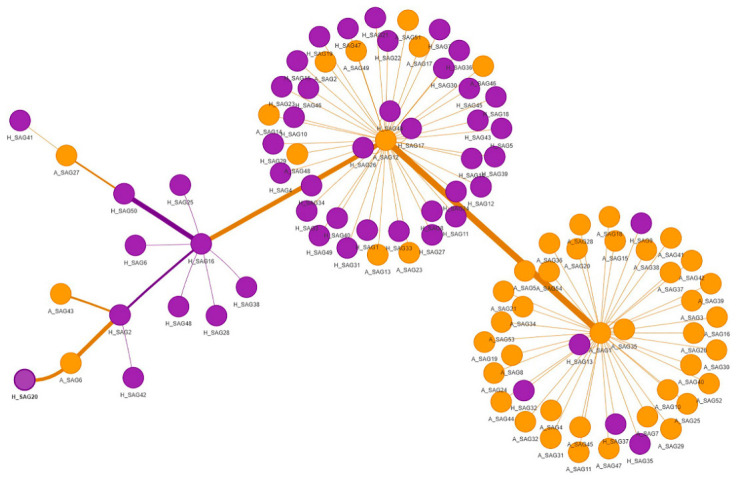
Minimum-spanning tree based on binary WGS-predicted AMR profiles of *S. agalactiae*, constructed from the presence/absence of predicted non-susceptibility to 12 antimicrobial molecules using Jaccard distance coloured by host origin (orange, animal-derived A_SAG; purple, human-derived H_SAG). The network reveals three major clusters: two large, densely connected groups (one human predominant and one animal predominant), and a smaller, more heterogeneous cluster linking highly divergent human and animal SAGs through long branches. The thick, elongated edges connecting the three clusters highlight deep evolutionary splits that may correspond to distinct clonal complexes or lineage groups.

A minimum-spanning tree (MST) was generated from binary AMR profiles based on ResFinder-derived WGS predictions of susceptibility/resistance to 12 antimicrobial molecules, encoded as presence/absence of predicted non-susceptibility, using Jaccard distances between isolates (Figure 7). In this network, each node represents a strain and edges are weighted by the Jaccard distance, so that shorter edges connect isolates with more similar resistance profiles, while longer edges link strains with more divergent combinations of resistances. Edge thickness in the figure reflects this underlying distance and is therefore interpreted strictly as a measure of relative similarity in AMR patterns, without any assumption about recent transmission or evolutionary timing.

The MST shows three main groups of closely connected isolates, broadly consistent with the patterns observed in the core-genome analyses, with one large cluster enriched in human strains, one predominantly bovine cluster, and a smaller, more heterogeneous group containing both host origins. We use this MST only as a descriptive visualisation of how multidrug-resistant profiles are distributed across lineages and hosts, while inference on evolutionary relationships and potential host-associated structure relies primarily on the core-genome phylogeny. This MST is used as a descriptive representation of similarity in multidrug resistance patterns and does not constitute a phylogenetic or transmission analysis.

PCA was applied to provide a synthetic multivariate view of the in silico AMR profiles. Unlike single-antibiotic prevalence comparisons, PCA allows the simultaneous evaluation of resistance patterns across all tested molecules, highlighting the main gradients of variation among isolates, the antimicrobial classes contributing most to this variation, and the presence of multidrug-resistant outliers.

The PCA analysis of antibiotic resistance profiles (Supplementary Figure S3A) shows that the first two principal components capture 87.1% of the total variance (PC1 = 59.3%, PC2 = 27.8%), indicating that the multidimensional resistance space can be effectively summarised in two axes.

PC1 is dominated by tetracycline and minocycline, which together account for almost half of the variance on this axis (loading ≈ 0.48 for both), whereas all other antibiotics contribute only marginally, highlighting tetracycline resistance as the main driver of separation among strains along PC1. PC2 is instead shaped primarily by the lincosamides lincomycin and clindamycin (loadings ≈ 0.30 each), with aminoglycosides and dalfopristin providing smaller but non-negligible contributions (loadings ≈ 0.08–0.09), while macrolides (erythromycin, azithromycin, spiramycin) do not contribute to either component because resistance to these molecules is invariant across isolates. In the biplot, the vectors for tetracycline/minocycline are long and oriented almost orthogonally to those of lincomycin/clindamycin, emphasising that variation in tetracycline resistance differentiates one subset of genomes, whereas lincosamide resistance discriminates another along PC2, with aminoglycoside and dalfopristin vectors of intermediate length radiating between these two directions.

The UpSet plot (Supplementary Figure S3B) complements these results by quantifying the prevalence and co-occurrence of resistance combinations in the dataset. The most frequent pattern is concurrent resistance to azithromycin, spiramycin and erythromycin, which is present in all strains and therefore represents the baseline macrolide resistance background. Among multidrug profiles, the largest intersection (44 isolates) corresponds to combined resistance to macrolides plus tetracycline and minocycline, whereas a smaller subset (14 isolates) additionally carries resistance to lincomycin and clindamycin, and only a few strains (n ≤ 6) show more complex patterns that include dalfopristin and/or aminoglycosides. Aminoglycoside resistance (streptomycin, amikacin, kanamycin, neomycin) is rare and typically confined to one or two highly resistant genomes in which it co-occurs with lincosamides, macrolides and tetracyclines, consistent with the long aminoglycoside vectors and the position of these outliers in the PCA space.

### 3.5. Resistance Genes

**Figure 8:**
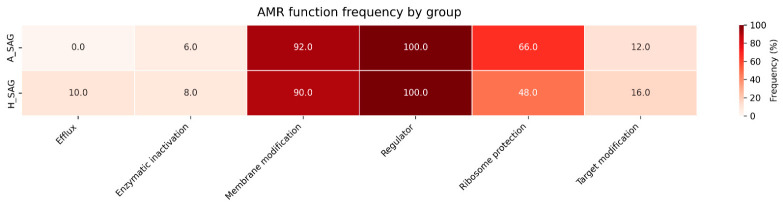
Heatmap showing the relative frequency of antimicrobial resistance functional categories in the A_SAG and H_SAG groups. Values within cells represent the percentage of AMR functions assigned to each category within the corresponding group. Peptide resistance/cell-envelope-associated functions were predominant in both A_SAG and H_SAG, accounting for 92.0% and 90.0% of the respective functional profiles. Aminoglycoside-, MLS_B- and tetracycline-associated functions occurred at substantially lower frequencies.

The distribution of AMR functional categories (Figure 8) was broadly similar between the A_SAG and H_SAG groups. Functions associated with peptide resistance and cell-envelope modification were predominant in both groups, accounting for 92.0% of the AMR functions detected in A_SAG and 90.0% in H_SAG. Aminoglycoside-associated functions were slightly more frequent in A_SAG than in H_SAG (4.0% versus 2.0%), whereas MLS_B-associated functions were more frequent in H_SAG (6.0%) than in A_SAG (2.0%). Tetracycline-associated functions represented 2.0% in both groups. Overall, the two groups showed a largely comparable AMR functional profile, dominated by functions related to peptide resistance and cell-envelope processes. Within the MLS_B category, *ermB* and *ErmA*/MLS_B were the predominant acquired determinants, frequently co-occurring with *lsaC* and *lnuC*, which mediate resistance to lincosamides and streptogramins and may contribute to broader MLS_B non-susceptibility backgrounds in both host groups.

### 3.6. Virulence Factors

**Figure 9:**
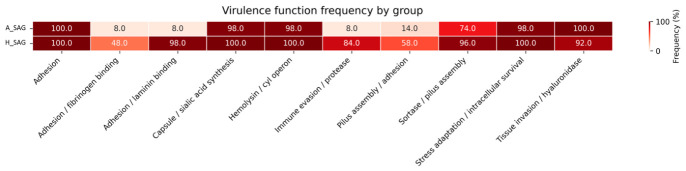
Heatmap showing the prevalence of virulence-associated functional categories in the A_SAG and H_SAG groups. Cell values represent the percentage of isolates within each group carrying at least one virulence determinant assigned to the corresponding functional category. General adhesion factors were detected in all isolates, while capsule/sialic acid synthesis, haemolysin/cyl operon and stress adaptation/intracellular survival functions were nearly ubiquitous in both groups. H_SAG showed higher frequencies of fibrinogen- and laminin-binding factors, immune-evasion/protease functions, and pilus- and sortase-associated determinants.

Figure 9 shows the distribution of virulence factors functional categories. These virulence-associated genes were identified by in silico screening against VFDB and represent genomic predictions only, without direct evidence of gene expression, functionality, or clinical impact (Supplementary Table S4). Accordingly, the presence of these loci should be interpreted as potential, rather than demonstrated, virulence traits.

Virulence-associated functional categories were widely represented in both A_SAG and H_SAG, although marked differences were observed for several functions. General adhesion determinants were detected in 100% of isolates in both groups. Capsule/sialic acid synthesis, haemolysin/cyl operon and stress adaptation/intracellular survival functions were also highly prevalent, occurring in 98% of A_SAG isolates and 100% of H_SAG isolates. H_SAG showed substantially higher frequencies of laminin-binding adhesion factors (98% versus 8%), immune-evasion/protease functions (84% versus 8%), pilus assembly/adhesion factors (58% versus 14%), fibrinogen-binding adhesion factors (48% versus 8%) and sortase/pilus-assembly functions (96% versus 74%). Conversely, tissue invasion/hyaluronidase-associated functions were slightly more frequent in A_SAG than in H_SAG (100% versus 92%). Overall, H_SAG displayed a broader prevalence of several adhesion-, pilus- and immune-evasion-related functional categories, whereas functions related to general adhesion, capsule production, haemolysis and stress adaptation were nearly ubiquitous in both groups.

Network analysis (Supplementary Figure S4) allowed for the identification of associations between specific resistance genes and virulence. At the center of the network are the *mprF* and *tet(M)* genes, which act as major hubs by connecting to numerous virulence factors. Particularly interesting is the distribution of the *cyl* family genes (*cylABDEFGIJKXZ*), which encode components of the β-hemolysin/cytolysin system, showing a strong connection with *mprF*. Quantitative co-occurrence analysis also reveals a significant differentiation between the resistance-virulence profiles of human and animal strains. While H_ isolates show a higher prevalence of associations between *tet(O)* and *pilC/pilA/srtC3/srtC4*, A_ strains exhibit unique connections between *rpoB2* (rifampicin resistance) and metabolic genes like *whiB3*. In this context, the co-occurrence network is intended as a descriptive representation of the simultaneous presence of AMR and virulence genes within the same genomes, in order to highlight recurring genetic profiles. Accordingly, while some AMR–virulence combinations appear recurrent, the present network analysis is purely descriptive and does not establish coevolution, co-selection, or mobile-element-mediated linkage between these traits. Such inferences would require additional analyses of genomic context, including gene colocalization on the same mobile genetic elements or chromosome regions, as well as formal statistical tests of association.

## 4. Discussion

### 4.1. Main Discussion

This study’s results offer essential insights into the comparative genomics and antibiotic resistance patterns of *S.agalactiae* (Group B *Streptococcus*, GBS) isolated from bovine and human sources in Lombardy, emphasizing the One Health implications of interspecies genotype and resistance determinant overlaps.

The pan-genome analysis confirms the classification of GBS as an organism genetically characterized by an “open” pan-genome, with significant variability in gene content (e.g., shell and cloud fractions) which facilitates accessory gene-mediated adaptation and host specificity [3]. The distinct segregation of phylogenetic clades, predominantly consisting of either animal or human strains, each exhibiting unique resistome and virulome signatures, indicates significant host-adaptive selection, despite the hypothetic potential for sporadic horizontal gene transfer events suggested by the identification of intermediate or mixed clades. These findings reflect recent European and international genomic monitoring indicating that, despite widespread genetic exchanges, niche-driven selection persists in promoting different evolutionary pathways in GBS [7,8]. The core component of the pan-genome reflects the essential functions required for bacterial survival and replication, whereas the accessory and unique gene clusters underpin phenotypic variability and the capacity to adapt to diverse environmental and immune conditions. The presence of 1109 core genes and 223 soft core genes suggest that, despite the great variability, there is a highly conserved set of essential functions.

The phylogenetic tree also reveals the presence of strains in intermediate positions or within mixed clades, which is consistent with, but does not demonstrate, possible spillover events between animal and human hosts. Nevertheless, the predominance of ‘pure’ clades indicates that such events could probably be rare. An additional relevant observation is the correspondence between phylogenetic structure and the distribution of resistance and virulence determinants. Clades composed predominantly of human isolates (H_) are frequently associated, in the network analyses, with a higher prevalence of resistance genes such as *tet(O)* and with pilusassociated loci (*pilC*, *pilA*, *srtC3/4*), as well as virulence determinants that are typical of adaptation to the human host. In contrast, animal clades (A_) show preferential associations with genes such as *rpoB2* and *whiB3*, supporting the existence of host-specific genomic signatures within the *S. agalactiae* population.

The overlap of STs (e.g., ST12, ST23) among isolates from humans and cattle expands epidemiological findings indicating that these clones operate as host generalists, able to colonize and spread in various mammalian hosts [17]. In alignment with earlier research, particularly from Denmark and the United Kingdom, which identified significant STs such as ST1, ST23, and ST103 in both bovine and human populations [8,9,17], our findings are compatible with a scenario in which interspecies transmission may be facilitated by direct or environmental interactions and by the adaptive versatility encoded in the accessory genome, although such processes cannot be formally demonstrated with the available data [10]. ST103, mostly linked to bovine milk worldwide, is also found in human isolates, reinforcing the hypothesis of possible spillover between cattle and humans, as evidenced by case reports [7]. This suggests that there is a dominant clonal population in animal hosts that is likely shaped by herd-level transmission and limited genetic influx. Indeed, synthesizing serotype and ST percent frequencies reveals that the human reservoir harbors both hypervirulent and diverse lineages, most notably the serotype III–ST17 combinations, whereas the animal reservoir points to overwhelming dominance by the serotype II–ST591 axis and limited diversification. The detection of less represented serotypes within our dataset, including serotype IV, should be interpreted in light of the current epidemiology of human GBS. Although serotype IV was not among the dominant serotypes in our collection, it should not be considered globally uncommon, as recent studies have reported its increasing involvement in human GBS infections, including lineages associated with high-level gentamicin resistance determinants. Therefore, its occurrence further supports the need for continued genomic and antimicrobial-resistance surveillance across human and animal reservoirs. The presence of not determined ST for both serotype and ST shows how incomplete genotyping is a problem in epidemiological surveys and the importance to obtain a full molecular characterization of isolated strains.

The WGS-predicted resistance profiles identified in this study indicate a concerning yet globally uniform background in which most strains harbour MLS_B determinants such as *ermB* and *ErmA*/MLS_B, together with *lsaC*/*lnuC* and related loci, consistent with predicted non-susceptibility to macrolides (spiramycin, erythromycin, azithromycin) and tetracyclines in both reservoirs. However, these findings represent genotypic indicators of potential non-susceptibility rather than phenotypically confirmed resistance, and thus should be interpreted with caution in the absence of AST data [3,36]. This pattern is particularly concerning, as macrolides represent one of the main therapeutic alternatives for treating *S. agalactiae* infections in patients with betalactam allergy. In contrast, tetracycline resistance (tetracycline and minocycline) shows a clear host-related trend, with almost universal non-susceptibility in human isolates and substantially lower levels in animal strains. This aligns with genomic epidemiology results from several European and worldwide contexts, indicating that excessive or unregulated antibiotic use in clinical and agricultural sectors has facilitated the selection and persistence of MDR GBS strains [7,8]. The observation that multidrug resistance patterns are shared by identical or closely related sequence types, irrespective of host origin, is consistent with a role of mobile genetic elements in facilitating horizontal transfer and dissemination of resistance determinants among *S. agalactiae* populations. The uneven yet overlapping distribution of resistance to lincomycin, clindamycin, and dalfopristin in both animal and human strains may reflect differences in local or host-specific antimicrobial usage, as suggested by previous genomic studies, although this remains speculative in the absence of detailed treatment data. The presence of resistance genes to dalfopristin in A_SAG isolates is particularly difficult to interpret, given that this antibiotic, as well as the quinupristin–dalfopristin combination, is not approved for use in cattle. A plausible explanation is that these genes have been acquired via mobile genetic elements originating from other co-circulating microbial populations exposed to different antimicrobial regimens, but their precise source and selection context cannot be determined from the current dataset [13,37]. This supports the hypothesis that such resistance might happen without direct selective pressure. For instance, an Iranian investigation found that more than 60% of *S. agalactiae* isolates from pregnant women were resistant to quinupristin-dalfopristin, even though this antibiotic was not used in cattle or humans in that area [38]. The authors explained this outcome with the presence of a lot of transferable resistance genes, including *ermB* and *tetM*, that are commonly found on mobile genetic elements that can move among bacteria [38]. Another study has shown mobile determinants like *lsa(C)* that confer lincosamides, streptogramins, and pleuromutilins cross-resistance [39]. The fact that they are present in unrelated *S. agalactiae* clones shows even more the hypothesis that they were acquired through horizontal gene transfer and not just clonal spread. Moreover, all A_SAG isolates are susceptible to beta-lactams, which is important because this is also what is shown in current literature and international surveys. Recent studies from Argentina, Thailand, and Italy all found that *S. agalactiae* field isolates are very unlikely to be resistant to beta-lactams. In fact, almost all of them were sensitive to penicillin and other beta-lactams. Indeed, *S. agalactiae* is less able to get exogenous beta-lactam resistance genes than other pathogens, explaining why this species is still susceptible. Historically, this makes beta-lactams the best treatment for bovine mastitis caused by this bacterium [5,40,41]. In a recent work by Massella et al. (2025), resistance to quinupristin/dalfopristin in resistant vancomycin enterococci strains and/or enterococci isolated from raw milk was, however, highlighted [42].

Hierarchical clustering and PCA underscore two pivotal characteristics (i) the predominant influence of tetracycline and lincosamide resistance in elucidating the variance among isolates, and (ii) the equilibrium of intrapopulation homogeneity alongside interpopulation diversity, which is further reflected in the symmetrical distribution of genomic and resistance traits [3]. The aggregation of ST1, ST17, and ST196, exhibiting common resistance combinations throughout both host groups, corresponds with recent evolutionary research that recognizes these STs as especially proficient in gene acquisition and ecological bridging [8]. Conversely, more “specialized” or “host-restricted” sequence types (e.g., ST380 in cattle and ST8 in humans) exhibit limited resistance profiles, reinforcing the notion of ecological specialization and adaptive divergence after host transitions [6,7].

Our findings corroborate earlier research indicating the correlation of significant virulence factors (e.g., *hvgA*, *Rib*, and the main pilus island gene content) with certain lineages and host backgrounds. The prevalence of *hvgA* and *PI-1/PI-2a* gene clusters in human, particularly neonatal-infecting isolates, corresponds with existing literature linking these genes to increased invasive potential and severe clinical outcomes in neonates [8]. In contrast, the animal-adapted lineages exhibit specific virulence and antibacterial factors, perhaps influenced by the unique immunological, nutritional, and pharmacological stressors present in the dairy environment [7,17].

The existence of accessory resistance genes, including *tet(O)*, *pilC*, *pilA*, and *srtC3/4* in human clusters, and *rpoB2* and *whiB3* in animal clusters, emphasizes the ability of the accessory genome to encode both resistance and niche-adaptive functions [3]. The presence of genes like *ErmA/B* (macrolide resistance) linked to multiple virulence factors, including capsular genes (*neu*) and adhesion genes (*pil*, *fbsA/B*), suggests a coevolution of pathogenic traits and antimicrobial resistance, potentially facilitated by mobile genetic elements such as transposons or shared plasmids. From a clinical standpoint, the co-occurrence of aminoglycoside resistance genes (*APH(3′)-IIIa*, *ANT(6)-Ib*, *aad(6)*) with key *S. agalactiae* virulence determinants such as *lmb* (laminin-binding protein) and *scpA/B* (C5a protease) is particularly concerning. This genetic finding suggests the emergence of strains that combine enhanced adhesion, tissue invasion and immune evasion with reduced susceptibility to aminoglycoside-based therapies, a combination that may favor persistent infection, therapeutic failure and more severe clinical outcomes [43,44,45].

The efficacy of comprehensive control programs, is well established and supported by the results in areas where regular monitoring, biosecurity measures have resulted in significant reductions in GBS incidence [2,17]. This epidemiological success indicates that herd-level surveillance and intervention are important for reducing the circulation of *S. agalactiae* in dairy populations and for limiting the emergence and dissemination of MDR lineages with One Health relevance [7,9]. Nonetheless, the enduring presence of genetically related multidrug-resistant bacteria in both groups indicates that the veterinary and clinical sectors require harmonized policies, especially for antimicrobial stewardship, resistance gene surveillance, and monitoring of high-risk clonal complexes.

### 4.2. Study Limitations

This study has some limitations that should be considered when interpreting the results. First, the human dataset was not prospectively matched to the bovine collection by time, geography, sampling frame, sequencing platform, or clinical/epidemiological metadata. Human genomes were retrieved from public repositories, which may introduce database-related biases, including uneven sampling, overrepresentation of clinically relevant or outbreak-associated lineages, variable assembly quality, and incomplete metadata. Although all genomes were analysed using a harmonized downstream workflow, residual batch effects cannot be fully excluded. Second, host-associated genomic differences may be partly confounded by lineage composition, because the bovine dataset was dominated by ST591, whereas the human dataset was more heterogeneous. Therefore, host-associated clustering should be interpreted as dataset-level genomic structuring rather than definitive evidence of host adaptation. Third, the study does not include direct epidemiological links, contemporaneous human–animal sampling, or within-farm human exposure data; consequently, the detection of shared sequence types or overlapping AMR/virulence determinants cannot demonstrate recent interspecies transmission or infer its directionality. Finally, AMR and virulence profiles were inferred in silico from genomic databases and were not validated by phenotypic antimicrobial susceptibility testing or functional assays. These predictions are useful for comparative screening but may not fully reflect gene expression, regulatory effects, resistance phenotype, virulence potential, or clinical relevance.

## 5. Conclusions

This study offers more evidence that *S. agalactiae* should be considered a dynamic, adaptable One Health pathogen, with its population structure influenced by a balance between core genome stability and accessory genome-driven innovation. Our findings underscore the necessity for integrated, cross-sectoral strategies for AMR monitoring, persistent molecular and phenotypic characterization of GBS populations, and sustained vigilance against the emergence and dissemination of MDR and hypervirulent lineages. A One Health approach is essential to prevent both current and future veterinary and clinical hazards posed by this genetically diverse organism. Although these findings support the One Health relevance of comparative genomic surveillance, they should be interpreted in light of the study limitations described above.

## Data Availability

All newly generated genome sequences were deposited in the National Center for Biotechnology Information (NCBI) under BioProject PRJNA1298752. The genome-level metadata (host, country, serotype, ST, and assembly metrics), the pan-genome gene presence–absence table, the AMR gene presence–absence matrix and the virulence gene presence–absence matrix are provided as Supplementary Tables S1, S2, S3 and S4 respectively. Other raw data used for this study can be obtained upon request to the corresponding authors.

## Supplementary Materials

The following supporting information can be downloaded at: https://www.mdpi.com/article/10.3390/ani16142257/s1.
