# Supplementary material for: Comparative Genomic Analysis of Bovine and Publicly Available Human Streptococcus agalactiae Genomes
Source: Animals (Basel). 2026 Jul 21;16(14):2257. doi: 10.3390/ani16142257 (PMC13405221; doi:10.3390/ani16142257)
Supplement: Supplementary file 1 [file animals-16-02257-s001.zip › animals-4424692-supplementary.pdf]

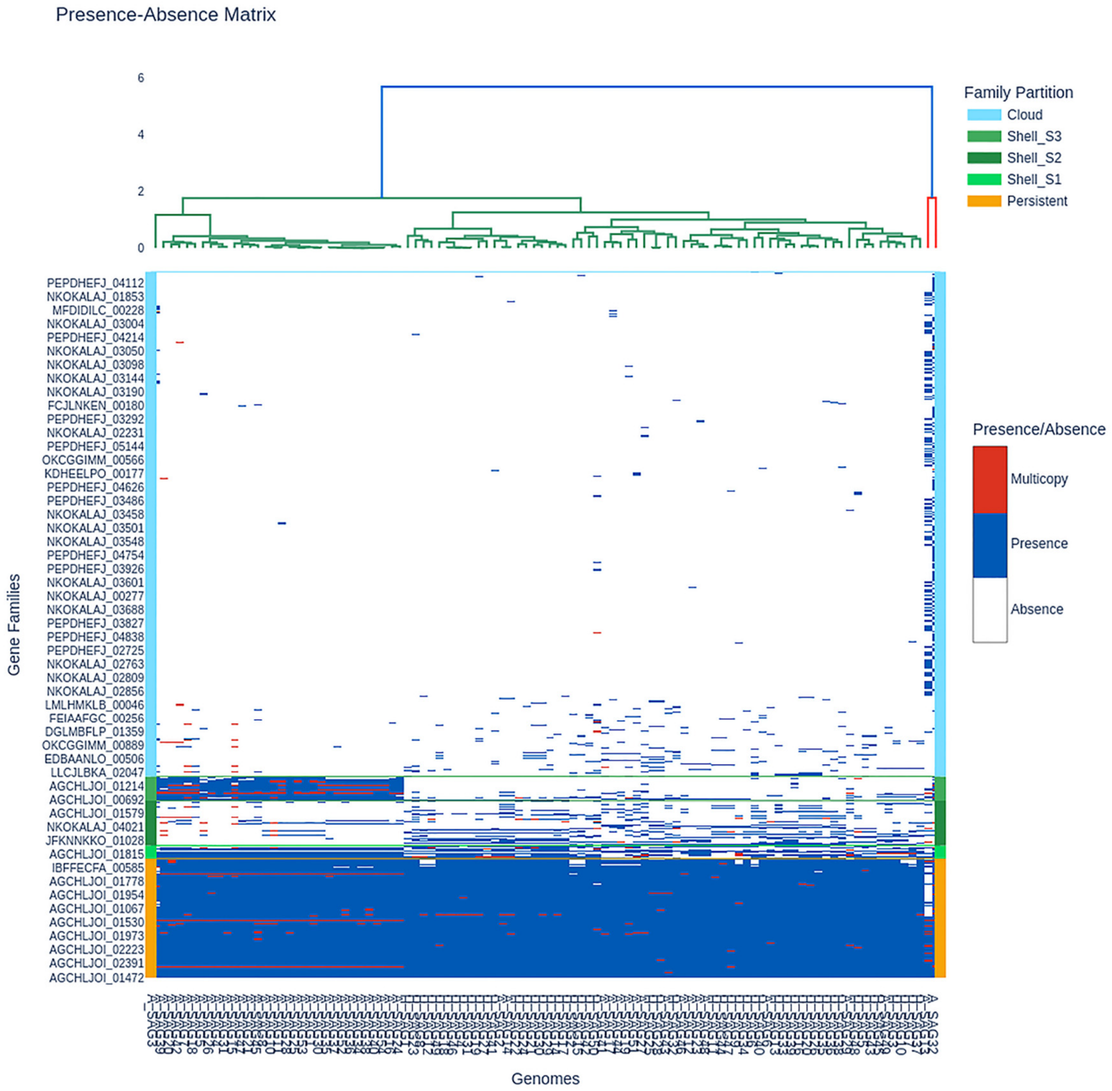

Supplementary Figure S1. Presence–absence matrix of *S. agalactiae* gene families. Heatmap of gene family presence, absence, and multicopy occurrence across the S. agalactiae genomes, as defined by PPanGGOLiN partitions (cloud, shell, and persistent). Hierarchical clustering of genomes based on their gene family content highlights structured variation within the accessory genome, whereas persistent genes form a dense block of universally conserved families along the matrix.

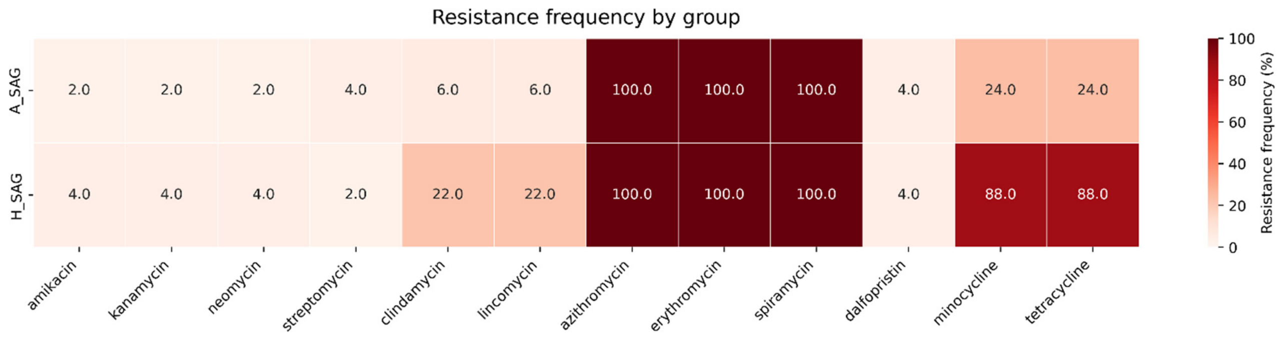

Supplementary Figure S2. Heatmap of predicted phenotypic resistances in human and animal isolates. Cell values represent the percentage of predicted non susceptible strains within the corresponding group.

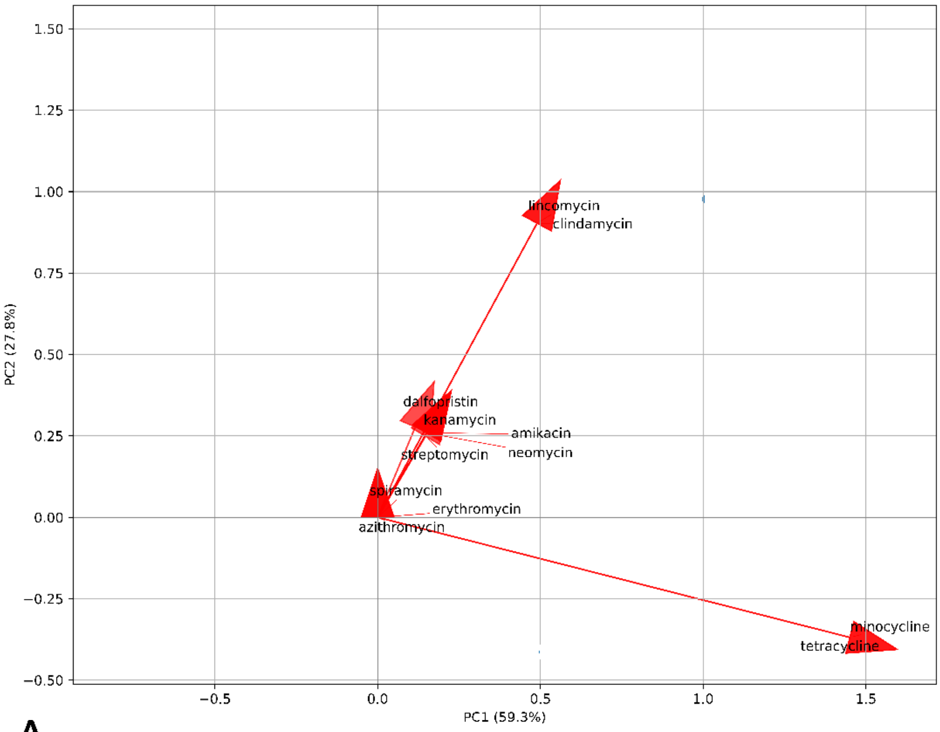

A

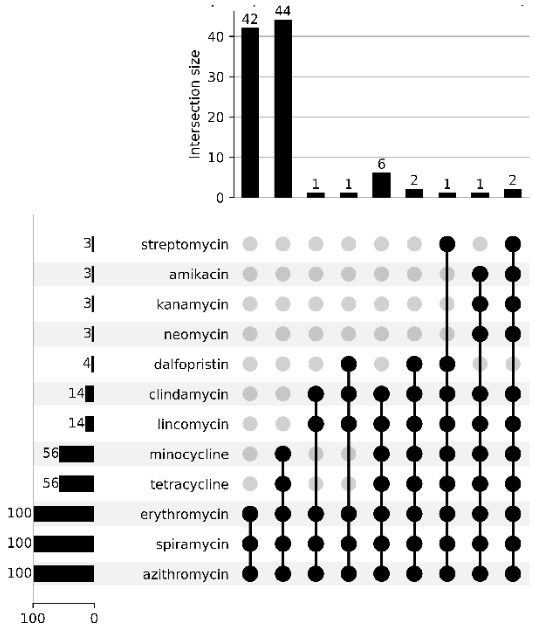

B

Supplementary Figure S3. Principal component and combination patterns of antimicrobial resistance in *S. agalactiae* single-amplified genomes. (A) Principal component analysis of binary resistance profiles to 11 antibiotics in *S. agalactiae* single-amplified genomes. The first two components explain 87.1% of the total variance (PC1 = 59.3%, PC2 = 27.8%), with PC1 mainly driven by resistance to tetracycline and minocycline and PC2 largely influenced by lincomycin and clindamycin. (B) UpSet plot showing the frequency of resistance combinations across the same genomes. Universal macrolide resistance (azithromycin, spiramycin, erythromycin) represents the dominant background, whereas co-resistance to tetracyclines and lincosamides, and more rarely to aminoglycosides and dalfopristin, defines a smaller subset of highly multidrug-resistant strains.

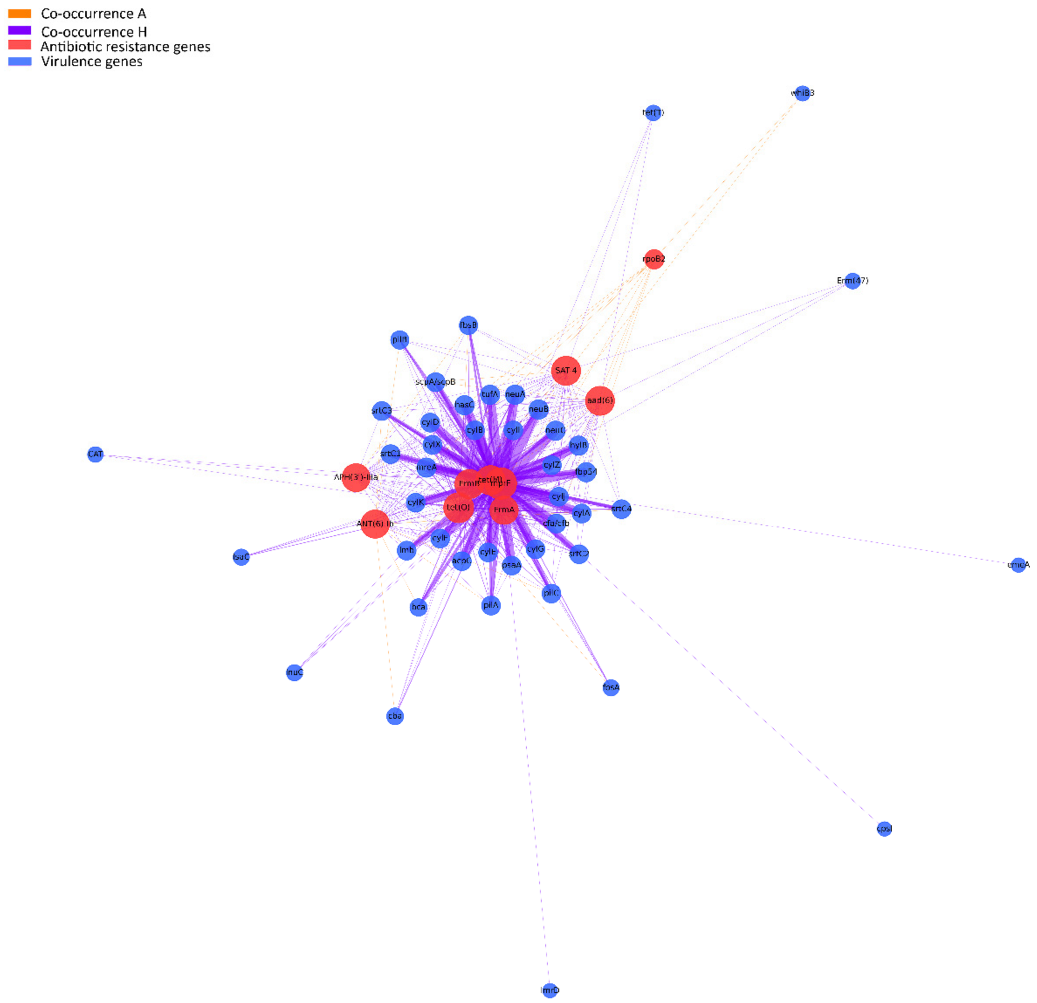

Supplementary Figure S4**.** Network of co-occurrences between antibiotic resistance and virulence factors.
