# Supplementary figures and images for: Comparative Genomic Analysis of Bovine and Publicly Available Human Streptococcus agalactiae Genomes
Source: Animals (Basel). 2026 Jul 21;16(14):2257. doi: 10.3390/ani16142257 (PMC13405221; doi:10.3390/ani16142257)

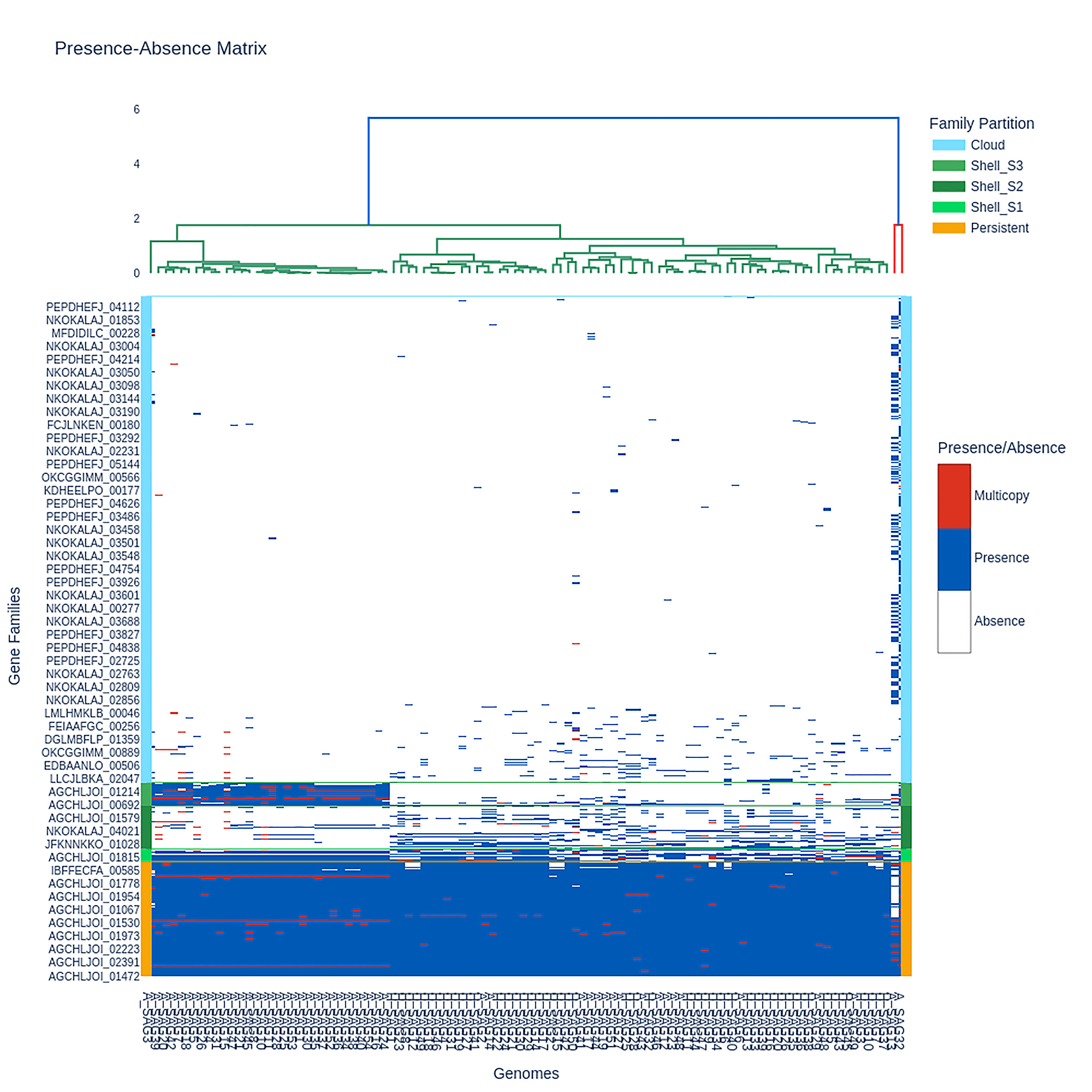

Supplement: Supplementary file 1 [file animals-16-02257-s001.zip › Supplementary Figure S1.png]

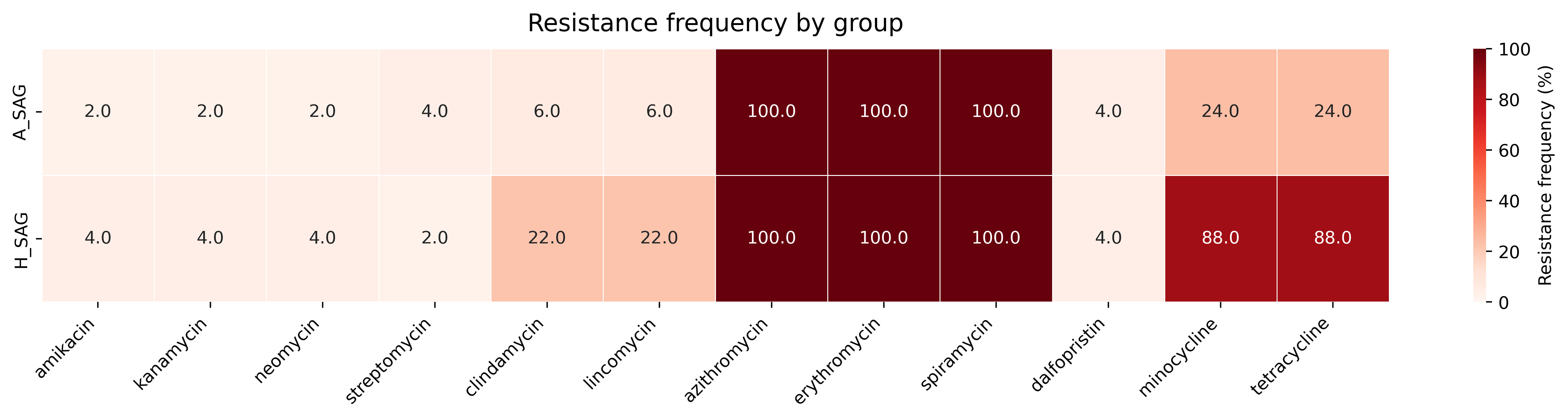

Supplement: Supplementary file 1 [file animals-16-02257-s001.zip › Supplementary Figure S2.png]

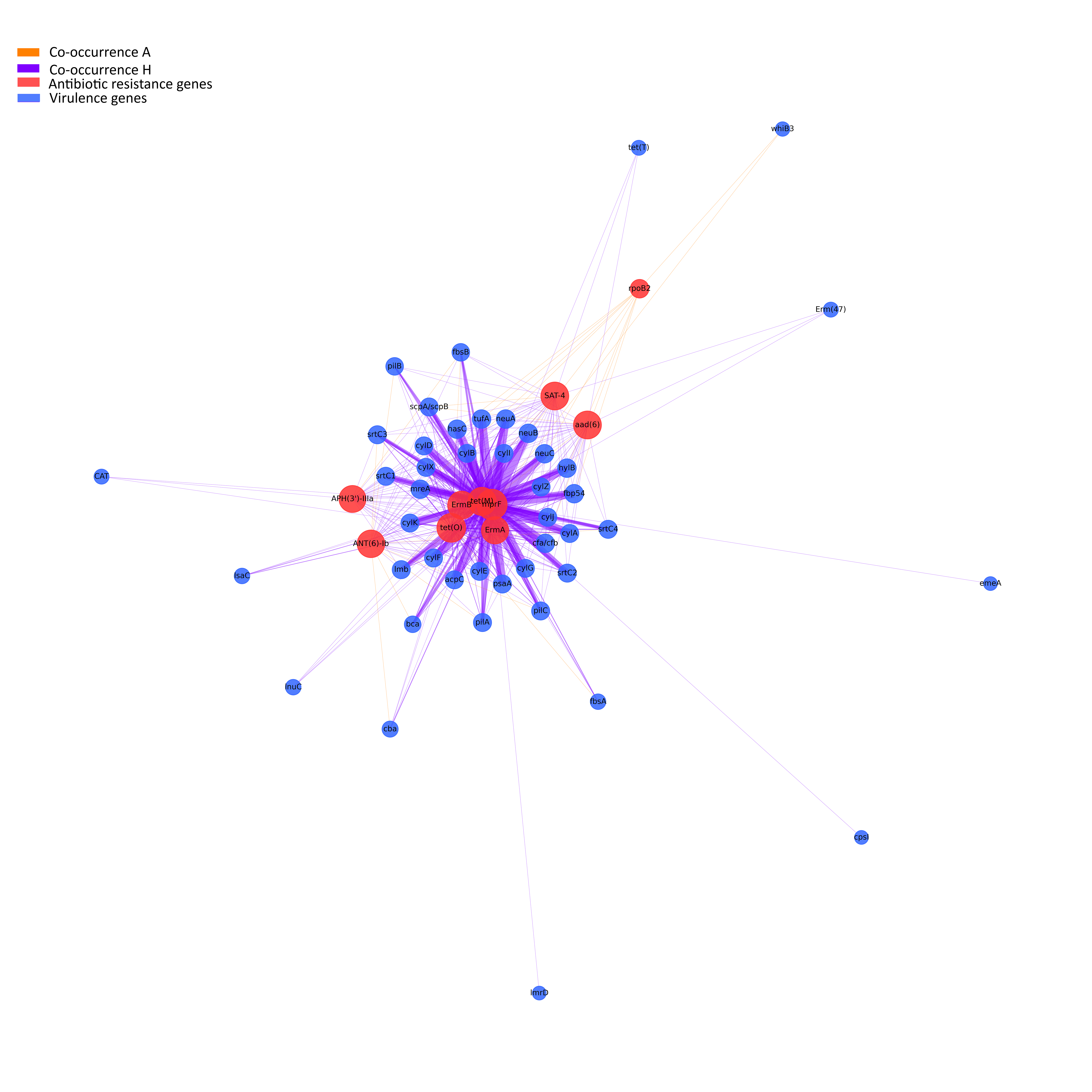

Supplement: Supplementary file 1 [file animals-16-02257-s001.zip › Supplementary Figure S4.png]
